# [Retracted] Clinical significance of bromodomain-containing protein 7 and its association with tumor progression in prostate cancer

**DOI:** 10.3892/ol.2026.15457

**Published:** 2026-01-12

**Authors:** Yong Liang, Baiping Dong, Jiangwei Shen, Caosheng Ma, Zhongping Ma

Oncol Lett 17: 849–856, 2019; DOI: 10.3892/ol.2018.9665

Following the publication of the above paper, it was drawn to the Editor's attention by a concerned reader that the majority of the panels shown for the cell migration and invasion assay experiments in Fig. 4C and D on p. 854 contained overlapping data, suggesting that these data were derived from a much smaller number of original sources than the diversity of experiments portrayed in this figure would have demanded; moreover, most of these data had also already appeared in an article written by different authors at different research institutes that was published previously in the journal *Oncotarget*.

Owing to the fact that the contentious data in the above article had already been published prior to its submission to *Oncology Letters*, the Editor has decided that this paper should be retracted from the Journal. The authors were asked for an explanation to account for these concerns, but the Editorial Office did not receive a satisfactory reply. The Editor apologizes to the readership for any inconvenience caused.

